# Deep Learning Approaches for the Prediction of Protein Functional Sites

**DOI:** 10.3390/molecules30020214

**Published:** 2025-01-07

**Authors:** Borja Pitarch, Florencio Pazos

**Affiliations:** Computational Systems Biology Group, National Center for Biotechnology (CNB-CSIC), 28049 Madrid, Spain; borja.pitarch@cnb.csic.es

**Keywords:** protein function, protein functional site, deep learning

## Abstract

Knowing which residues of a protein are important for its function is of paramount importance for understanding the molecular basis of this function and devising ways of modifying it for medical or biotechnological applications. Due to the difficulty in detecting these residues experimentally, prediction methods are essential to cope with the sequence deluge that is filling databases with uncharacterized protein sequences. Deep learning approaches are especially well suited for this task due to the large amounts of protein sequences for training them, the trivial codification of this sequence data to feed into these systems, and the intrinsic sequential nature of the data that makes them suitable for language models. As a consequence, deep learning-based approaches are being applied to the prediction of different types of functional sites and regions in proteins. This review aims to give an overview of the current landscape of methodologies so that interested users can have an idea of which kind of approaches are available for their proteins of interest. We also try to give an idea of how these systems work, as well as explain their limitations and high dependence on the training set so that users are aware of the quality of expected results.

## 1. Introduction

The relative ease in obtaining the amino acid sequences of proteins contrasts with the difficulty in finding functional information for them. Thanks to modern sequencing technologies [1], today, it is possible to obtain the sequences of an organism’s repertory of proteins (proteome) by sequencing its genome. It is also possible to obtain the sequences of millions of protein and protein fragments from a metagenomics or environmental sample with a complex mixture of uncharacterized organisms [2]. Nevertheless, associating functional information to these sequences, either globally (i.e., infer the function of a given protein) or locally (which residues/regions of a protein are important for different aspects of its function), is not so straightforward. As a consequence, most proteins for which we know the amino acid sequence are not functionally characterized. Even in the well-curated UniProt database [3], less than 10% of the proteins have some kind of associated functional information [4], a figure that is much lower than that of other less annotated protein sequence databases. The functional characterization at the residue level is one order of magnitude lower (~1%).

The experimental characterization of a protein’s functional sites is slow and expensive, as it basically involves mutating every residue of the protein and assaying the effect on its function. Such experimental approaches cannot cope with the increasing deluge of newly sequenced proteins. Nevertheless, knowing which residues in a protein are related to its function is very important, not only to obtain insight into it from a molecular point of view but also to devise ways of modifying it for our benefit [5]. Consequently, much effort was put into developing methodologies for predicting a protein’s functional regions from the wealth of available sequence information [4,5,6].

Most of these approaches are sequence-based. They take as input multiple sequence alignments of homologous proteins and look for position variation patterns related to functionality, such as full conservation, subfamily-dependent conservation, or co-evolving positions [6]. While these sequence-based methods have existed for a long time, they are still highly used as they take advantage of the abundant sequence information, are quite reliable, and complement other more sophisticated methodologies.

There is also a plethora of methodologies that use structural information for detecting functional regions, normally in combination with the sequence-based approaches commented on earlier. These approaches have been recently boosted by the development of deep learning-based methodologies. In fact, deep learning-based AlphaFold [7] or RosettaFold [8] are successful in predicting accurate three-dimensional (3D) structures, which are comparable to experimental structures. These approaches can generate high-quality models for virtually every sequenced protein. Notably, the developers of AlphaFold were awarded the 2024 Nobel Prize in Chemistry.

As in many other areas in bioinformatics, in the area of functional site prediction, there was a progression in the methodologies from statistics to “classic” machine learning and now to deep learning. Deep learning is a class of machine learning approaches (systems that learn from examples to relate inputs and outputs) that use large neural networks with complex architectures and many internal layers. As in human activities in general, and in research in particular, deep learning is revolutionizing inference tasks for which large enough datasets are available for training [9,10]. These methodologies are not only assisting the prediction of functional sites through the generation of accurate 3D structural models, commented on in the previous paragraph, but there are deep learning-based methods specifically trained for this task as well.

In this review, we aim to provide an overview of the main deep learning-based approaches for predicting protein functional regions. We use a general definition of “functional site”: any residue whose modification affects any functional aspect of the protein without affecting the structure. Hence, this definition includes active sites, binding sites, post-translational modifications, non-functional variants related to diseases, etc., although methodologies are generally focused on a particular type of functional site. This review does not try to be comprehensive but covers only some representative examples of the many approaches used in this area to give readers an idea of the main tendencies of the field.

This review does not cover the prediction of global protein function (i.e., molecular activity or cellular role of the whole protein) and only focuses on the prediction of functional residues. The prediction of global protein function using deep learning approaches is also a hot topic and has been covered in other excellent reviews (e.g., [11]). The target audience of this review is composed by life scientists, potential users of these methodologies. Hence, the technical details of these complex methods are kept to a minimum, although a primer on deep learning, references, and tables with further information are included.

## 2. A Primer on Deep Learning

Deep learning is a very complex area involving many technical aspects. It is impossible to give an overview of the field at any depth here. Nevertheless, a basic description of the main concepts can help users of these methodologies to understand what they have in their hands, as well as their capabilities and limitations. For a deeper introduction to the subject, the reader is referred, for example, to [9].

**Figure 1:**
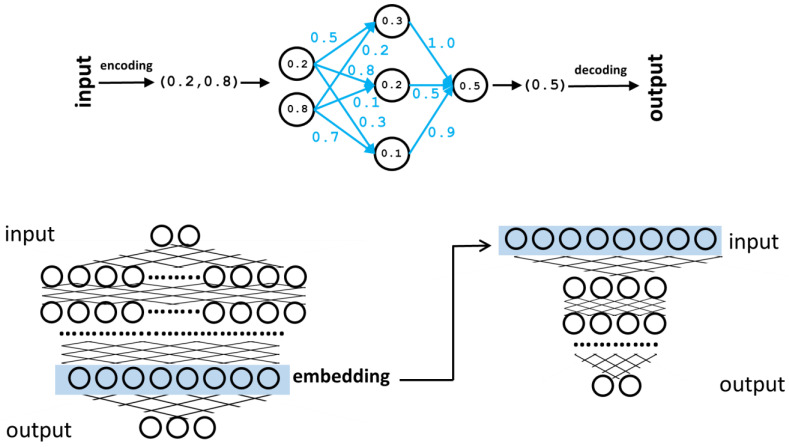
Neural networks. Top: representation of a simple neural network with one hidden layer. The input is encoded as a two-component vector. These input values are “propagated” through the network, taking into account the weights of the connections (blue) to finally generate the output vector (a single value in this case). During the training phase, the weights (blue) are tuned to maximize the matching between the generated outputs and the real ones of the training set. Bottom: schematic representation of a larger neural network with many hidden layers and internal neurons. One of these layers (light blue box) can be taken as an alternative coding for the input vectors (embedding) and used, among other things, as input for another neural network (right) trained for a different task (“transfer learning”).

Machine learning methods are systems designed to carry out inference tasks without explicit instructions or a procedural algorithm. They do that by learning from solved examples of that task. A set of examples is shown to the system (“training”), and it gets ready for processing a new case not seen in its training set (“prediction”). Neural networks are a class of machine learning systems designed to relate numerical inputs and outputs (i.e., predict the output for a given input) when that relationship is complex and cannot be expressed by an explicit mathematical function. Their architecture and functioning try to resemble what we know about the information processing by the human brain. The inputs and outputs are coded as vectors of real numbers, whose components are depicted as “neurons” in the standard graphical representation of these systems (Figure 1). Apart from the input and output layers of artificial neurons, these systems have one or more internal layers, termed “hidden layers”. The neurons of a given layer are connected to those of the next layer, and these connections have associated numerical values representing their strengths (weights). The values of the input layer neurons are determined by the input vector. The value of a given neuron in the forthcoming layers is given, in the simplest case, by the sum of the values of the neurons connected to it in the preceding layers multiplied by the corresponding weights (Figure 1). In most cases, more complex “activation functions” are applied to calculate these values, eventually involving thresholds, again trying to replicate how the activation of biological neurons works. So, with this procedure, starting with a given input vector, a final output vector is generated. During the training process, a set of known input–output pairs of vectors is shown to the network, and the weights of the connections (which start with random values) are adjusted to minimize empirical risk: the difference between the predicted output and the actual target vector. After this training process, the weights of the connections should have values optimized to relate inputs and outputs, at least those in the training set. With these trained weights, the network is ready to “predict” (generate an output vector for an unseen input). Note that in a neural network without hidden layers, the values of the output neurons can be calculated from the inputs with a simple linear function. The objective of the architecture of hidden layers is to approximate complex non-linear functions with many parameters (weights of the connections) relating to inputs and outputs.

Although described in a very simplified way here, the training process is complex and a critical part of the development of a predictor. The training set has to be well balanced in terms of examples and try to uniformly cover the spaces of possible inputs and outputs the network is going to face later at the prediction stage. If the training set is biased to a particular class of examples, the network will be “too adapted” to that class and will not generalize well for others (overfitting). Besides being well balanced, the training set has to be as large as possible, especially in deep learning systems, which require a lot of training examples in order to optimize the many parameters they have

“Deep” neural networks differ from these “classic” neural networks in their size (they have many more internal layers and hence neurons) as well as their architecture, which deviates from that succinctly described above and is more complex and adapted to specific tasks. Table 1 contains descriptions of some deep learning architectures and approaches widely used for making predictions on biological data. For example, the well-known ChatGPT system for generating human-like text in response to a prompt is a deep neural network with a “transformer” architecture (Table 1) and hundreds of billions of connections. The input of ChatGPT is a sequence of “words”, and the output is a ranked list of possible words that would fit as a continuation of that sequence, sorted by a probability. Starting with the user prompt, iterative applications of the network to the growing chain of words generate the text we see. ChatGPT was trained with a large corpus of texts to optimize these hundred billion parameters. This particular class of deep learning systems trained to predict the next item in a sequence are called language models, and they are very successful learning features of biological sequences (“biological language model”) due to their intrinsic sequential character [12].

It has been shown that, in a neural network already trained for some objective, the vectors represented by some internal layers, even if not having an evident “translation” into the “objects” originally coded in the input layer, contain modified representations of them that can be useful for other tasks. These internal vectors are called embeddings [12] (Figure 1). They can be used as they are, for example, for clustering input objects based on this new vectorial representation; the clustering generated is better than the equivalent cluster of the original input vectors concerning the problem the neural network is solving. However, they can also be used as input for another neural network trained for a different task, which is termed “transfer learning” (Figure 1). In transfer learning, during the training phase, the first network (previously trained for another task) is only used for re-coding the inputs; its weights are not changed, and only the weights of the second network are tuned for its new task.

## 3. Deep Learning Approaches for Predicting Protein Functional Sites

**Figure 2:**
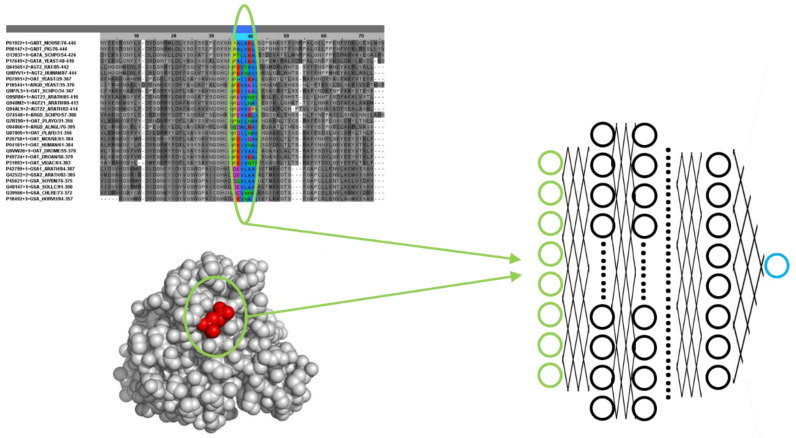
Example of a deep neural network setup for predicting functional sites. The input layer of the network (green) codes a set of sequence and/or structural features of a particular protein residue, such as amino acid type, evolutionary pattern in a multiple sequence alignment, neighbor residues in 3D, solvent accessibility, etc. The output layer is, in this case, a single neuron (blue) whose value represents whether the residue is functional (e.g., “1.0”) or not (“0.0”).

There is an enormous variety of machine learning and deep learning approaches for predicting protein functional sites from sequence and/or structural information. Figure 2 schematizes an example of a possible simple deep neural network setup for this task. The input layer of the network codes for different structural and/or sequence features of a particular protein residue. The sequence features usually come from a multiple sequence alignment, as these are rich sources of functional information [13] and represent, for example, the evolutionary pattern of that residue (conservation level, amino-acid variations, etc.). Eventually, it can also include that information for a sequence window around the residue of interest or even the whole alignment to include long-range information (on co-varying positions, for example). At first, most methods tried to use sequence information alone as that is abundant. But now that it is possible to generate high-quality structural models for virtually any protein thanks to AlphaFold and similar systems, most methods also include structural information in one way or another. The structural information coded in the input layer for the residue of interest can include, for example, its amino acid type, those of other residues close to it in the 3D structure, solvent accessibility, secondary structure, distances to other residues, etc. The output layer codes for whether that residue is functional or not, for example, with a single neuron coding “1” for functional and “0” for non-functional. The neural network is trained with (many) examples of functional and non-functional residues (from the same and different proteins) according to the definition of “functional” we are interested in (e.g., active sites, binding sites, post-translational modifications, all, etc.). As commented earlier, during this training process, the network optimizes its internal parameters to maximize the matching between the numerical codification of the features of a residue (input) and its functional character (output). After this training, the network is ready to make a prediction for a new residue.

In the following, we describe some representative examples of deep learning-based approaches for predicting functional and active protein sites. These are also listed in Table 2, together with links to the corresponding source code or web server. Table 1 contains short descriptions of the main deep learning architectures and approaches they use.

The most obvious impact of deep learning on protein functional studies is through the high-quality structural models generated by AlphaFold [7] and similar methods, which shine light on many protein structural and functional aspects as they can model the (virtually) whole protein universe [14,15,16]. In many cases, a 3D protein structure alone gives clues about possible functional sites (e.g., pockets, tunnels, certain secondary structure motifs associated with function, known 3D arrangements of residues in active sites, etc.). Consequently, AlphaFold is predicting functional sites indirectly. For example, AlphaFold has been shown to be a good predictor of disordered regions [17], and these protein segments are usually protein binding sites. Improved versions of the program can also predict the structure of multimers (hence pointing to protein binding sites) and protein–ligand complexes (ligand binding sites and active sites).

Transmembrane helices can also be inferred from deep learning-generated structural models (previous paragraph), for example, looking for α-helices with a specific length and hydrophobicity pattern. But there are also deep learning approaches specifically designed for this task, such as DeepTMpred [18]. There are also predictors for the other type of membrane-spanning structures, the beta-barrels, e.g., BetAwareDeep [19], as well as predictors able to deal with both types of motifs at the same time [20].

There are many works focused on the generation of general-purpose embeddings (see above and Figure 1) for protein local sites. These embeddings are then used for various downstream tasks (“transfer learning”), including the prediction of different kinds of functional features. For example, COLLAPSE [21] uses as input structural features (atoms close in 3D to the residue of interest) as well as evolutionary data (multiple sequence alignments) to generate embeddings coding for a residue and its structural neighborhood. These embeddings are then used to predict protein interaction sites, sites whose mutation changes the stability of the protein, and PROSITE [22] functional motifs. COLLAPSE embeddings can also be used outside prediction scenarios to compare sets of residues (e.g., similarity between the active sites of two proteins).

Some deep learning methods aimed at predicting a protein’s global function can also take those predictions to a local or even residue level thanks to their internal architecture, hence effectively predicting functional sites. For example, the method developed by Jang et al. uses sequence information alone as input for predicting protein function, and the contribution of each residue to that function can be inferred, hence generating also predictions at the residue level [23]. DeepFRI is another predictor of protein function that uses sequences and structures as input [24]. It is able to “zoom in” the representation from the protein to the region level, allowing the detection of sites related to specific functions. PARSE [25] uses the embeddings of local structural features generated with COLLAPSE (commented above) in a transfer learning framework for predicting enzyme functions and can also take these predictions to the residue level, effectively detecting the associated catalytic sites.

ScanNet uses structural information as input and, representing atoms and amino acids, including 3D neighborhood information, effectively predicts protein binding sites in these structures [26]. The program is available through a web server. Another method based on structural information uses a very general representation (raw atom distributions) to predict functional sites represented by PROSITE motifs or catalytic sites [27].

A particular type of functional residues, the post-translational modifications (PTMs), are targeted by an important number of deep learning predictors. For example, Zhu et al. developed a predictor of different PTMs (phosphorylation, acetylation, and ubiquitination) that uses as input the sequence, structure, and dynamical features [28]. The dynamical information is a distinctive characteristic of this predictor, and interestingly, it is shown to contribute largely to the predictive performance of some PTMs. Other examples of PTM predictors are DeepNphos [29], for predicting N-phosphorylation sites, and NetGPI [30], for predicting glycosylphosphatidylinositol anchoring sites.

Another specific type of functional site, the ligand binding site, has been targeted by many works due to its importance for drug binding. For example, [31] predict drug binding sites with a deep learning approach that uses sequences as input, while the predictor developed by [32] uses structural information and was trained to predict ligand binding sites in general, not only those for drugs.

There are also many predictors dealing with protein local features related to protein targeting and sorting [33], usually using sequence information as input, as these features are quite evident at that level. For example, SignalP 6.0 predicts all types of signal peptides using protein language models [34], and TargetP predicts transit peptides, determining the sorting of the protein to different subcellular compartments [35]. Cellular localization predictors usually report the protein region responsible for the targeting. Within these, we can distinguish “two-class” predictors, trained to discriminate whether a protein is going to a particular compartment or not (e.g., [36]), and “multi-class” predictors, able to select among a number of compartments in a single prediction shot (e.g., [37]).

A plethora of deep learning methods have been developed for predicting the effect of amino acid changes (mutations) on protein stability and interactions, mainly with the aim of interpreting disease-associated variants and locating interface hot spots [38]. These can also be considered functional site predictors as these residues are important for different aspects of the protein’s function. For example, DDMut-PPI [39] predicts the effect of mutations on protein interfaces and can be used through a web server. ProMEP [40] uses sequence and structural information to predict mutation effects. The method developed by Brandes et al. uses a protein language model to predict variant effects, and it is fast enough to scan the genome for millions of possible variants [41]. Cagiada et al. trained a predictor on a large dataset of known variant effects and showed how it could effectively predict functional sites of different natures (active sites, allosteric sites, binding sites, etc.) [42]. Some methods are tailored to the prediction of the effect of mutations on a specific type of function, such as EnzyACT, which predicts the effect of mutations on enzyme activity and hence can be used as a predictor of active sites [43].

**Table 2:** Representative deep learning methods for predicting protein functional sites. All URLs were accessed on 6 December 2024.

Name	Prediction Goal	URL(s) and/or Reference	Deep Learning Architecture/Approach
AlphaFold	Protein 3D structure	https://alphafoldserver.com/	Deep neural network with a diffusion generative model
		[7]	
DeepTMpred	Transmembrane helices	https://github.com/ISYSLAB-HUST/DeepTMpred	Combination of different DL models
		[18]	
BetAware-Deep	Transmembrane beta barrels	https://busca.biocomp.unibo.it/betaware2	Deep recurrent neural network
		[19]	
TMbed	Transmembrane helices and barrels	https://github.com/BernhoferM/TMbed	Embbedings of protein language models
		[20]	
PhiGnet	Protein function and associated functional sites	https://doi.org/10.5281/zenodo.12496869	Graph neural networks
		[23]	
DeepFri	Protein function and associated functional sites	https://beta.deepfri.flatironinstitute.org/	Convolutional neural network and pre-trained embedding with protein language models
		[24]	
PARSE	Enzyme functions and associated catalytic sites	https://github.com/awfderry/PARSE	COLLAPSE embeddings [21] and traditional statistics
		[25]	
ScanNet	Protein binding sites	http://bioinfo3d.cs.tau.ac.il/ScanNet/	Geometric deep learning
		[26]	
--	PROSITE motifs [22] and catalytic sites	https://simtk.org/projects/fscnn	3D Convolutional neural network
		[27]	
cDL-PAU, cDL- FuncPhos	Different post-translational modifications	https://github.com/ComputeSuda/PTM_ML	Combination of different neural networks
		[28]	
DeepNphos	N-phosphorylation sites	https://github.com/ChangXulinmessi/DeepNPhos	Convolutional neural network
		[29]	
NetGPI	Glycosylphosphatidylinositol anchoring sites	https://services.healthtech.dtu.dk/service.php?NetGPI	Recurrent neural network
		https://github.com/mhgislason/netgpi-1.1	
		[30]	
DeepConv-DTI	Drug targets and drug-binding residues	https://github.com/GIST-CSBL/DeepConv-DTI	Convolutional neural network
		[31]	
DeepDrug3D	Ligand binding sites	https://github.com/pulimeng/DeepDrug3D	Convolutional neural network
		[32]	
SignalP	All five types of signal peptides	https://services.healthtech.dtu.dk/service.php?SignalP-6.0	Protein language models
		https://github.com/fteufel/signalp-6.0	
		[34]	
TargetP	Transit peptides	http://www.cbs.dtu.dk/services/TargetP-2.0/	Recurrent neural network
		https://github.com/JJAlmagro/TargetP-2.0/	
		[35]	
SCLpred-EMS	Sorting signal to endomembrane/secretory pathway	http://distilldeep.ucd.ie/SCLpred2/	Convolutional neural networks
		[36]	
DeepLoc	Sorting signals for different subcellular compartments	https://services.healthtech.dtu.dk/service.php?DeepLoc-2.0	Protein language models
		[37]	
DDMut-PPI	Effect of mutations on protein interactions	https://biosig.lab.uq.edu.au/ddmut_ppi	Graph convolutional neural network
		[39]	
ProMEP	Effect of mutations	https://github.com/wenjiegroup/ProMEP	Protein language models
		[40]	
ESM1b	Disease variant effect	https://github.com/ntranoslab/esm-variants	Protein language model
		[41]	

## 4. Example

**Figure 3:**
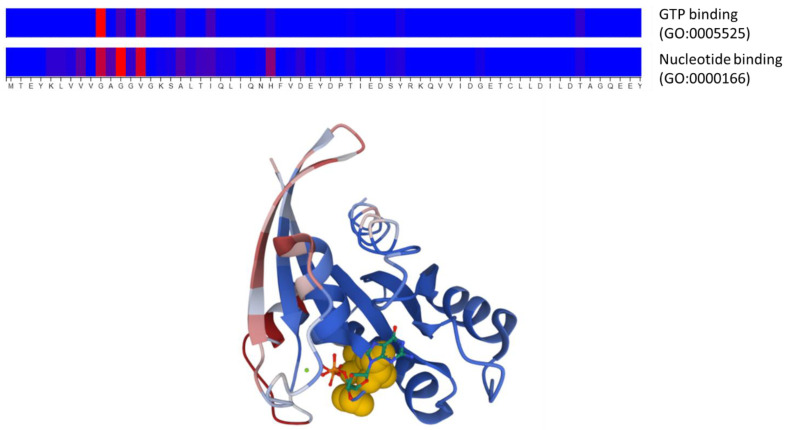
Example of a prediction of functional sites and protein binding sites from sequence and structure information using two deep learning approaches. The top panel shows the functional sites associated with two Gene Ontology molecular function terms (“nucleotide binding” and “GTP binding”) predicted by DeepFRI [24] for the human RasH protein, using its sequence as the only input. The color scale represents the prediction score, from blue (low) to red (high). Only the N-terminal part of the protein, where the top predictions are, is shown. The three residues with the highest scores (G10, G12, and V14) are also highlighted in yellow in the 3D structure of this protein (lower panel), where the bound nucleotide (GTP) is shown in stick representation. The lower panel shows the prediction of protein binding sites generated with ScanNet [26] for this same protein using structural information as input, in a color scale going from bluish colors (low) to reddish (high). This 3D representation was generated by the Molstar molecular viewer (https://molstar.org/) integrated into the ScanNet server.

To illustrate the kind of predictions these methods generate and the input they take, we applied two of these approaches to the well-studied RasH human protein. This is a GTPase that acts in signal transduction, interacting with other proteins in a functional cycle driven by GTP binding and hydrolysis. We used the DeepFRI method [24] through its web server (Table 2) to predict the function(s) of this protein as well as the associated functional sites, in this case, using the sequence as input (UniProt accession: P01112). The server correctly predicts the molecular functions (Gene Ontology terms) known for this protein, “GTP binding” and “GTPase activity”, as well as their ancestors in the GO hierarchy (e.g., “nucleotide binding”, “small molecule binding”, etc.). Figure 3 shows the representation generated by the server of the predicted functional sites associated with two of these GO terms related to GTP binding. These residues clearly map around the known binding site for this nucleotide and comprise the well-described phosphate-binding loop (“P-loop”, at residues 10–17). To complement this prediction, we used another server, ScanNet [26] (Table 2), to predict protein binding sites for this protein using its 3D structure as input (PDB ID: 4q21). The predicted sites (Figure 3) include the “switch-I” and “switch-II” regions of RasH (residues 26–38 and 61–73, respectively), known to be involved in the interactions with effectors [44].

## 5. Discussion

The prediction of protein functional sites/regions has always been an important area in bioinformatics due to the importance of detecting such sites in protein sequences and structures. Many methodologies exist, and they are routinely applied to proteins of biomedical and biotechnological interest, usually in combination with experimental approaches aimed at confirming the predictions or implementing the proposed mutations for tuning or changing the protein’s function [5].

Among other techniques, classic machine learning methods such as support vector machines or neural networks were applied to this problem [45]. With the advent of deep learning, the field exploded; as for some functional aspects, the large amounts of available data were perfectly suited for these data-hungry systems. This is because the performance of deep learning can be impressive, but these systems require large amounts of data for training. As a rule of thumb, these methods require training a number of examples ten times the number of tunable parameters (i.e., number of connections) [46]. This precludes the usage of these systems for functional aspects for which not enough examples are available for training [33]. This is in part alleviated by “transfer learning” approaches, where a neural network pre-trained for a different problem (for which enough data is available) can generate embeddings that might work better than the original inputs in a simpler neural network trained for a second learning task for which fewer examples are available.

Besides the large amounts of protein sequences available for training and generating embeddings, their intrinsic sequential nature (forgive the redundancy) makes them very suitable for a successful class of deep learning approaches, such as language models. Indeed, revising the temporal trends of the landscape of deep learning methodologies for predicting protein functional sites, a tendency towards protein language models to the detriment of other architectures is evident [12,33]. Finally, a third factor that contributed to the adoption of deep learning (and machine learning in general) in this field is the quite trivial codification of protein sequences into vectors for feeding these systems. For other biological entities, such as chemical compounds, the codification is not so trivial, and some assumptions have to be made, which move away a little from the input vectors from the entities they try to represent.

Another problem with the datasets of functional residues used for training/testing is that they might be biased and/or not represent a protein’s whole repertory of functional sites. For example, the experimentally known binding sites for a particular protein depend on its known complexes, as well as the particular conformational states it presents on them. In this context, many “negatives” (residues considered non-functional) might not be such.

It can be seen that most of these problems will be alleviated when more experimental information (both in size and diversity) becomes available for training these methods.

Although the predictive performance of these methods is excellent, an important drawback compared with other approaches is their intrinsic “black box” nature. This means that, while they predict functional sites with high reliability, in general, it is not possible to obtain either the knowledge on how they made these decisions or the explicit rules to make the predictions out of the network itself. In other words, they do not provide scientific knowledge on functional sites. This is a general problem for neural networks. For example, we can say that AlphaFold is able to “fold” proteins in silico (a long-sought goal in bioinformatics), but we learned nothing about the folding process itself.

As more methods are presented in the literature, independent initiatives for fairly benchmarking these systems using common datasets of functional features are required. This is particularly important for machine learning systems as they are prone to overfitting and lack of generalization, and consequently, the performance values calculated by the authors for a particular training/testing set might not hold for other independent datasets. In this sense, initiatives such as CAFA (Critical Assessment of Function Annotation) [47] are crucial. Establishing standards and good practices for training and evaluating deep learning approaches is also important [48,49]. In any case, as these approaches are based on different concepts and predict different types of functional residues, in general, it is advisable to run more than one method in order to combine the results, as illustrated with the RasH example.

Deep learning is also changing the field of protein design, with methods able to design new sequences compatible with a given fold, function, or able to bind a given molecule, as well as the other ways around [50]. A remarkable milestone in the field was the work of David Baker and his group on a deep learning-based methodology aimed at designing a protein scaffold able to “host” a desired functional site (arrangement of residues in 3D) given as input [51]. Although this can be considered just the opposite of what this review is about (i.e., knowing an active site predicts a protein sequence/structure for it), we include it here for its enormous importance for understanding protein functional sites and how to engineer them for our benefit.

Contrary to protein sequence and structure, which are univocal and well-defined concepts, “protein function” is less defined and difficult to quantify, depends on our available experimental toolkit, and, in many cases, is something we impose on proteins. This problem with the definition of protein function itself permeates to the definition/annotation of “functional site”, and probably our datasets of functional sites are biased or incomplete due to that. A more univocal definition of protein function and functional site, grounded on a molecular basis, as well as associated experimental approaches for determining them, will for sure improve the datasets of experimental functional sites and, consequently, the deep learning methods trained on them.

## Figures and Tables

**Table 1:** Deep learning architectures and approaches used by the described methods.

Type	Description
Transformer	Architecture specifically designed for processing data with a sequential character, mainly text. It can dynamically weigh the significance of different components of the input via a mechanism called “attention”.
Language model	Deep learning approach that, using transformers or other architectures, is trained to predict the next item in a sequence.
Protein language model	Language model that handles protein amino-acid sequences
Generative model	Approach designed to generate new data “similar” to those it was trained on. For example, generating “feasible” new protein sequences.
Recurrent neural networks	Neural network architecture in which the inputs and outputs are of the same nature and codified in the same way. Consequently, the output of a given iteration can be used as input for the next. They are used for processing sequential or temporal data, for example.
Graph neural network	Neural network whose architecture (neurons and connection) is not general but that of a graph representing the phenomenon the network is intended to model.
Convolutional neural network	Neural network architecture in which matrix operations are applied on some of its layers besides the typical forward propagation operations. Especially suited for handling spatial data, either 2D (e.g., images) or 3D (3D convolutional NN, e.g., three-dimensional structures)
Geometric deep neural network	Deep neural network architecture especially suited for handling generic geometrical data.

## Data Availability

No datasets associated to this review article.
